# Discontinuation Rates of Tadalafil Alone and in Combination with a-Blockers in the Treatment of Male Lower Urinary Tract Symptoms with or without Coexisting Erectile Dysfunction: A Systematic Review and Meta-Analysis

**DOI:** 10.1155/2022/9298483

**Published:** 2022-11-03

**Authors:** Qiang Chen, Yinjun Mao, Huiliang Zhou, Songxi Tang

**Affiliations:** ^1^Department of Andrology & Sexual Medicine, First Affiliated Hospital of Fujian Medical University, Chazhong Road No. 20, Fuzhou 350005, Fujian, China; ^2^Department of Pharmacy, First Affiliated Hospital of Fujian Medical University, Chazhong Road No. 20, Fuzhou 350005, Fujian, China

## Abstract

**Purpose:**

We examined the discontinuation rates of tadalafil alone and in combination with a-blockers (ABs) for the treatment of male lower urinary tract symptoms (LUTS), with or without erectile dysfunction (ED).

**Materials and Methods:**

We searched the EMBASE, PubMed, Web of Science, Scopus, Cochrane Library, and ClinicalTrials.gov databases for studies published until May 15, 2022. The discontinuation rates associated with LUTS medications were subsequently analyzed by meta-analysis.

**Results:**

Forty-four studies, including 1724 discontinued patients, were included. The combined discontinuation rate was 12.78% (95% confidence interval (CI) 9.89–15.98%), and the discontinuation rates because of adverse events and lack of efficacy were 4.56% (95% CI 3.39–5.90%) and 3.30% (95% CI 1.53–5.72%), respectively.

**Conclusions:**

The discontinuation rate of tadalafil alone or in combination with ABs for LUTS with or without ED was relatively low and varied according to the study type. Patients receiving monotherapy or combination therapy were similarly likely to abandon treatment. Treatment with a fixed-dose combination was associated with better persistence than with a free-dose combination. These data may help guide clinicians in selecting drug regimens when making decisions. Factors associated with treatment withdrawal need to be determined through high-quality clinical studies to reduce the drug discontinuation rate, which will ultimately reduce healthcare costs and improve patient outcomes.

## 1. Introduction

Several preclinical and clinical trials have demonstrated a strong correlation between erectile dysfunction (ED) and lower urinary tract symptoms (LUTS) [1], several of which have also shown that LUTS caused by benign prostatic hyperplasia (BPH) is an independent risk factor for ED [1]. Treatment approaches aim to avoid BPH-related complications, stop BPH progression, and improve symptoms and quality of life. The currently available medical therapies for LUTS include 5a-reductase inhibitors (5ARIs), a-blockers (ABs), and their combination therapy (CT) [2, 3]. Although effective, these treatments can exert side effects associated with sexual dysfunction [4].

Tadalafil, a phosphodiesterase type 5 inhibitor (PDE5i), has been shown to be effective in once-daily and/or on-demand treatment for ED, with proven efficacy in multiple controlled clinical trials, including those in LUTS patients with and without ED [5, 6]. Tadalafil acts directly on the micturition phases, not just penile erection, by increasing nitric oxide levels in the smooth muscle to relax the prostate and bladder neck [7, 8]. The efficacy of PDE5is in combination with ABs for remission of LUTS has also been assessed. Currently, research has shown this treatment to have beneficial additive effects on both sexual function and LUTS compared to monotherapy [9]. Hence, the opportunity to treat LUTS with or without ED by using tadalafil alone or in combination with ABs may lead to new and increasingly tailored treatment strategies.

However, several clinical surveillance studies have suggested that the discontinuation rates of medical treatment for LUTS, including overactive bladder or BPH, are quite high [10, 11]. In these studies, dissatisfaction and therapy failure were identified as the main reasons for drug withdrawal. To our knowledge, longitudinal information regarding the use of tadalafil alone or in combination with ABs in patients with LUTS is limited. Thus, we performed this systematic review and meta-analysis to investigate the discontinuation rate of tadalafil alone or in combination with ABs in male LUTS patients with or without ED.

## 2. Materials and Methods

### 2.1. Study Protocol

This study protocol was registered on the International Platform of Registered Systematic Review and Meta-Analysis Protocols (INPLASY, registration number: INPLASY202260105) [12].

### 2.2. Search Strategy

The English-language literature was systematically reviewed until May 15, 2022, in accordance with the criteria of the Preferred Reporting Project for Systematic Reviews and Meta-Analysis (PRISMA) [13]. The PRISMA checklist is provided in Table S1. The EMBASE, PubMed, Scopus, Web of Science, Cochrane Library, and ClinicalTrials.gov databases were searched to identify studies reporting the effects of tadalafil alone or in combination with ABs to treat LUTS patients with or without coexisting ED. We performed an extensive search using Medical Subject Headings (MeSH) terms and related keywords: “tadalafil,” “lower urinary tract symptoms,” “Cialis,” and “bladder overactive” (Table S2). Additionally, references in the retrieved articles were manually searched to attempt to identify any studies not found in the original literature search.

### 2.3. Study Selection

Based on the population, intervention, comparison, outcomes, and study design (PICOS) approach, the trials included in our study met the following criteria: (P) male patients with LUTS with or without ED; (I) consuming medication: tadalafil alone or in combination with ABs; (C) none; (O) evaluating the incidence of drug discontinuation: studies must report patients discontinuing medication, regardless of reasons; and (S) prospective, retrospective, observational, and randomized clinical trials (RCTs) were included. We placed no restrictions on the sample size, drug dose, or follow-up duration. We excluded any studies which were repeated publications, or those for which the original data for the literature could not be provided, or those for which the authors did not receive a response after contacting the authors. We further excluded any non-human studies, case series, case reports, commentaries, and reviews.

### 2.4. Study Outcomes

This study aimed to measure the discontinuation rate of the main treatment drug (tadalafil alone or in combination with ABs) for LUTS. Discontinuation was defined as the nonpersistence of the main treatment drug prescribed at the start of the first treatment, regardless of the reason. The discontinuation rate was calculated by dividing the number of patients who discontinued treatment by the total number of initial index patients. Further, the discontinuation rate due to adverse events (AEs) was defined to include only patients who discontinued treatment because of AEs in the numerator. Similarly, the discontinuation rate due to lack of efficacy was defined to include only patients who discontinued treatment because of drug inefficacy in the numerator.

### 2.5. Data Extraction and Quality Assessment

The authors CQ and M-YJ separately extracted data from each included study and crosschecked the results after extraction. Any disagreements between reviewers regarding data extraction were resolved through consensus discussion with a third reviewer. The information extracted from the included studies was as follows: (1) publication date, (2) name of the first author, (3) country of study, (4) type of design, (5) drug regimen and dose received by the patient, (6) number of participants per drug regimen, (7) treatment period, and (8) data on total number of patients who discontinued treatment, discontinuation due to AEs, and no efficacy.

Bias assessments of observational studies were rated on the Newcastle-Ottawa Scale, whereas RCTs were evaluated using the Cochrane risk of bias assessment tool.

### 2.6. Statistical Analyses

The statistical software R version 4.0.3 (package “meta”) was used for all statistical analyses.

Due to differences in the methodological and clinical perspectives in the included studies, we surmised that the heterogeneity among the included studies may be large. Hence, we used a random-effects model to obtain pooled estimates and a 95% confidence interval (CI) of the discontinuation rate, and the variance was stabilized by an arcsine transformation [14, 15]. Heterogeneity was assessed using Cochran's value and *I*^2^. The heterogeneity test was cut off at significant Cochran Q-values (*P* < 0.1) and *I*^2^ > 50%, as an *I*^2^ value of 30–50% was suggested as a cutoff value for moderate heterogeneity [16]. A prediction interval (PI) for the proportion in a new study was calculated if the argument prediction and comb. random were TRUE [17].

A cumulative meta-analysis was conducted to determine whether the discontinuation rate stabilized with an increasing sample size. A sensitivity analysis was further performed to phase out each study to test the reliability of the discontinuation rate.

In addition, subgroup analyses were performed according to drug regimen (monotherapy or CT), study design (prospective observational study, retrospective observational study, or RCT), and combination form (fixed-dose combination (FDC) (5 mg tadalafil/0.4 mg tamsulosin or 5 mg tadalafil/0.2 mg tamsulosin) or free-dose combination (different doses of tadalafil + different doses of Abs)).

## 3. Results

### 3.1. Search Results and Study Characteristics

**Figure 1:**
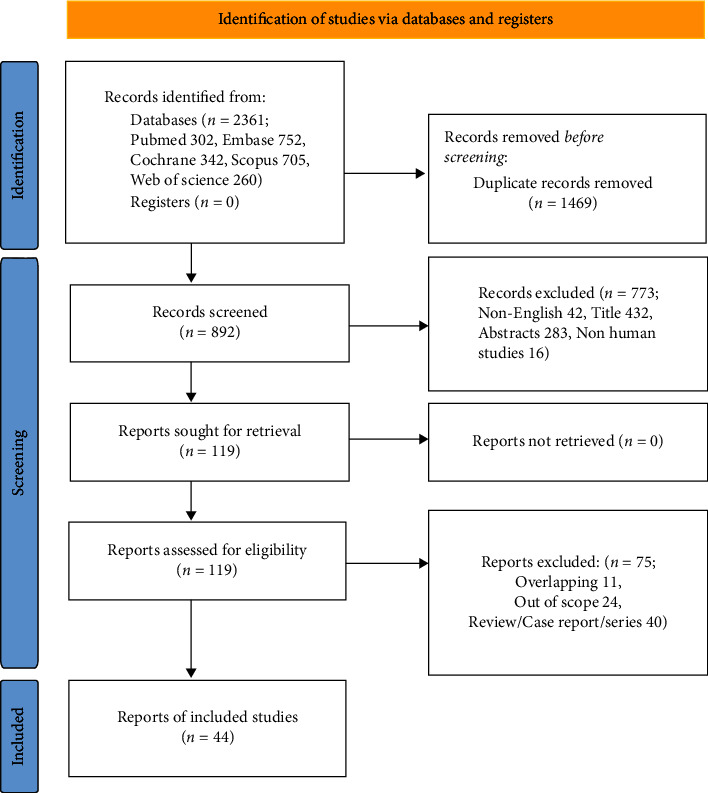
PRISMA flow chart illustrating the study selection process.

The initial literature search revealed 2361 studies, of which 44 comprising a total of 1724 discontinued patients were deemed eligible. The PRISMA literature selection flowchart is shown in Figure 1.

The included studies, published between 2007 and 2021, [5, 6, 18–59] included 11 prospective studies [27, 28, 35, 39–41, 43, 47, 50, 51, 58], 2 retrospective studies, [36, 37] and 31 RCTs. Thirty-four studies came from a single country, with Japan and Korea predominating, while the rest were multinational studies. Overall, 34 studies evaluated tadalafil monotherapy, 6 evaluated CT with ABs [30–36, 53], and 4 evaluated tadalafil monotherapy plus CT [31–33, 48]. One study used a combination of alfuzosin [48], 2 studies partially used a combination with alfuzosin or silodosin, [35, 53] 1 study used a combination with uroselective (alfuzosin, silodosin, or tamsulosin) and nonuroselective (doxazosin or terazosin) ABs, [54] and the rest used a combination with tamsulosin. The characteristics of the included studies are summarized in Table 1.

### 3.2. Quality Assessment

Tables S3 and S4 present a full summary of the risk of bias for the included studies. Of the 13 observational studies, seven (54%) were of high quality [35, 37, 39, 40, 43, 51, 58] and the rest were of moderate quality; 46% of the observational studies did not mention adjustment for confounders [27, 28, 36, 41, 47, 50]. The details of the 31 RCTs were as follows: (1) selection bias: 12 out of 31 studies reported the method of random sequence generation, [6, 23, 26, 31, 33, 34, 44–46, 49, 52, 55] while the others did not. Eight studies involved allocation concealment, [6, 23, 26, 33, 34, 46, 49, 53] while the remaining studies did not provide any information about allocation concealment; (2) performance and detection bias: 20 studies were double-blind, 2 were double-blind plus open-label, [25, 33] 1 was single-blind, [32] and 5 did not apply any blinding [31, 42, 45, 48, 55], while the remaining studies did not provide any information on blinding; (3) attrition bias: all studies provided information on the dropout rate or on patients lost to follow-up; and (4) reporting and other bias: all studies had a sufficient follow-up time (>1 month) to detect changes in quality of life or AEs. As such, there was no significant reporting or other bias.

### 3.3. Discontinuation Rate

**Figure 2:**
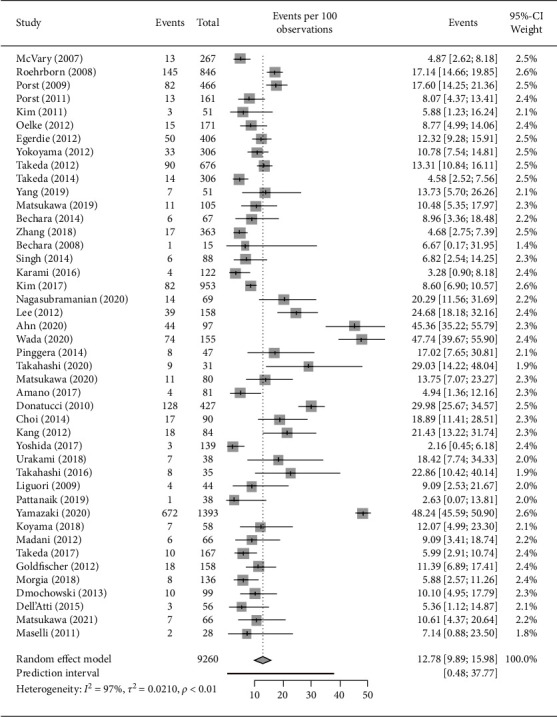
Forest plot illustrating the single study and summary incidence of discontinuation with tadalafil alone or in combination with ABs for LUTS with or without ED. ABs: a-blockers; CI: confidence interval; ED: erectile dysfunction; LUTS: lower urinary tract symptoms.

**Figure 3:**
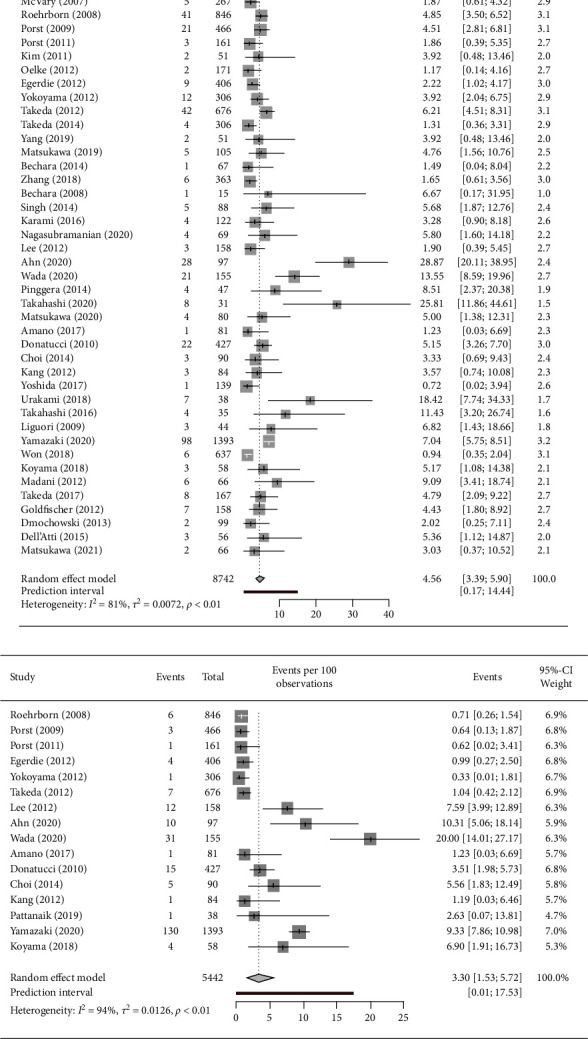
Forest plot illustrating the discontinuation rate due to AEs and no efficacy. (a) AEs, (b) No efficacy. AEs: adverse events; CI: confidence interval.

Figure 2 shows the discontinuation rate in each study and overall. The discontinuation rates in LUTS patients ranged from 2.16% to 48.24% in the 44 studies. The combined discontinuation rate was 12.78% (95% CI 9.89–15.98%), with a high heterogeneity (*I*^2^ = 97%), and the PI was 0.48 to 37.77. The overall discontinuation rate due to AEs was 4.56% ((95% CI 3.39–5.90%), I^2^ = 81%, PI (0.17–14.44), Figure 3(a)), whereas the discontinuation rate due to no efficacy was even lower (3.30% (95% CI 1.53–5.72%), *I*^2^ = 94%, PI (0.01–17.53), Figure 3(b)).

### 3.4. Subgroup Analyses of Discontinuation Rate

The results of the subgroup analyses for the discontinuation rate in patients with LUTS are summarized in Table 2 and Figure S1. Among the different types of study designs, the discontinuation rate in retrospective observational studies was higher than in other study designs (46.82% (95% CI 40.70–52.99%)), followed by prospective observational studies (17.53% (95% CI 10.87–25.39%)), and RCTs (9.78% (95% CI 7.66–12.12%)).

Patients undergoing CT (12.87% (95% CI 6.59–20.86%)) were similarly likely to discontinue monotherapy treatment (12.16% (95% CI 9.20–15.46%)). In contrast, the rate of treatment discontinuation caused by AEs in patients receiving CT (6.43% (95% CI 2.72–11.59%)) was higher than that in patients receiving monotherapy (4.12% (95% CI 3.08–5.30%)).

The treatment discontinuation rate was lower with free-dose combination therapy (10.62% (95% CI 5.84–16.61%)) than with FDC (24.00% (95% CI 0.52–66.34%)).

However, the opposite conclusion was reached when the only retrospective observational study (that of Ahn et al. [36]) was removed from the FDC (8.18% (95% CI 6.36–10.33%)) vs. free-dose combination (10.62% (95% CI 5.84–16.61%)) subgroup.

### 3.5. Cumulative Analysis of Discontinuation Rate

Cumulative meta-analysis results showed that, with an increase in sample size, point estimation tended to be stable and CI gradually decreased, indicating that the greater the sample size, the higher the accuracy of the results (Figure S2).

### 3.6. Sensitivity Analysis of Discontinuation Rate

When each study was sequentially excluded from the analysis, the sensitivity analysis results (12.08% to 13.14%) remained in good agreement with the discontinuation rate, indicating that the study results were robust (Figure S3).

## 4. Discussion

LUTS symptoms and ED severity tend to increase with age [60]. Hence, as with other chronic diseases, long-term use of LUTS and ED medications is important to improve the patient's symptoms and quality of life [61]. Although tadalafil alone and in combination with ABs has been proven to be effective and well tolerated in clinical studies, patient outcomes in clinical practice are not always consistent with research findings. One reason for this may be the relatively high rates of premature drug discontinuation [10]. This is the first report to investigate the discontinuation rate of tadalafil alone or in combination with ABs for the treatment of male LUTS with or without ED. The total discontinuation rate was 12.78%, of which the discontinuation rates due to AEs and ineffectiveness were 4.56% and 3.30%, respectively.

Overall, the discontinuation rates observed herein appear to be relatively low, despite the inclusion of observational studies and RCTs. Interestingly, the discontinuation rate was much higher in observational studies than in RCTs. The lower discontinuation rate in clinical studies may be due to both better adherence to recommendations made in the clinical trial setting and increased patient motivation [33]. Additionally, in clinical trials, patients are closely observed and receive medication free of charge during the study period [62]. This can improve the incidence of drug persistence. In contrast, in real-world clinical practice, patients often pay for drugs, which may result in higher expectations for their efficacy as well as a decreased tolerance for side effects. Moreover, a previous study also showed that self-reported economic status was related to long-term medication persistence [63]. In this study, respondents were asked whether their household income was sufficient to meet their needs. Persistence was 30% higher among those who answered “sufficient” or “more than sufficient.” Knowledge of these factors has allowed more illuminating clinical research to be conducted in real-world practice settings; hence, the actual incidence of treatment discontinuation can be calculated more accurately. Nevertheless, it is inevitable that the discontinuation rate will be underestimated in retrospective studies. The reasons for this include that patients who were excluded due to unrecorded follow-up examinations could not be analyzed, and it is not possible to know whether they discontinued treatment or continued treatment at another hospital. In some cases, discontinuation data are obtained through prescription records or self-reports rather than independent audits of pill counts or other, more accurate, verification methods. Treatment persistence may be overestimated if patients fail to refill their prescriptions.

Even more interestingly, patients who were treated with CT or monotherapy had similar discontinuation rates (12.87% vs. 12.16%). These data contradict the results of prior research evaluating long-term treatment for LUTS, which showed a lower discontinuation rate for CT (doxazosin + finasteride) (18%) compared to doxazosin (27%) and finasteride (24%) monotherapy [64]. In addition, the discontinuation rates reported in our study were relatively lower than those calculated in this study. However, it is difficult to compare the two studies owing to the differences in drug regimens and study design (meta-analysis vs. prospective randomized study), and these differences should therefore be interpreted with caution. Notably, discontinuation of tadalafil or ABs was easier and more regulated than that of 5ARIs or antimuscarinics (AMs). In fact, tadalafil and ABs both show rapid activity and can be easily and effectively retaken [26, 65].

However, compelling evidence in the literature suggests that treatment persistence is inversely correlated with the complexity of medication regimens. With this in mind, so-called FDC drugs (compilation of two or more drugs in a single tablet/pill) have been developed to treat multiple clinical conditions (e.g., hyperlipidemia and hypertension) or one disease (e.g., asthma, diabetes mellitus) in a complementary manner [66]. When only RCTs were included, a single tablet in an FDC regimen has a lower discontinuation rate than the use of two tablets in the free-dose combination for the treatment of LUTS. Our findings are consistent with the results from two retrospective observational cohorts of men with LUTS treated with AM + AB in Spain and the Netherlands, showing FDC had higher persistence rates than free-dose combinations [67, 68]. This advantage has been extensively demonstrated in other pathologies. A meta-analysis of hypertension data published between 2000 and 2017 showed that patients who received FDC had higher treatment persistence for their hypertension medication than those who received a free-dose combination [69].

Unlike the overall discontinuation rate, the discontinuation rate due to AEs was higher with CT than with tadalafil monotherapy. Although most AEs are self-limiting, they directly affect a patient's perception of treatment satisfaction. The least wanted undesirable effect was ejaculatory dysfunction (retrograde or diminished ejaculation), which is a well-known side effect of selective ABs [70]. This has previously been shown to be the main factor associated with high satisfaction in the tadalafil only group compared with the PDE5-I combination or tadalafil combination groups (both of which include the use of ABs) [71]. In our study, 9 patients in the CT group (tadalafil + tamsulosin) discontinued due to ejaculatory dysfunction. Interestingly, none received tamsulosin or silodosin before taking the CT [36]. However, other AEs related to tadalafil may have a smaller effect on drug withdrawal, in line with previous studies investigating once-daily tadalafil, which reported discontinuation rates due to side effects as low as 1% to 5% [72–74].

### 4.1. Limitations

This study had several limitations which should be mentioned. First, adherence/compliance with a therapeutic regimen or time to discontinuation has previously been identified as a public health issue that may exert a significant impact on clinical outcomes. However, a lack of concrete data makes it impossible to assess this indicator. Second, there were differences in the subjects' characteristics and demographics, dosing regimen, drug class, study population, entry criteria, study type, and study length, all of which may have led to the heterogeneity encountered between the studies, and may have affected the final results of the study. Therefore, these results should be interpreted with caution. Third, our results are subject to the limitation that the study did not include ABs monotherapy. However, it should be recognized that ABs are only applicable to the treatment of LUTS and not ED, which is a factor in the targeting of comorbid conditions. Finally, because of the inclusion of different study types, we did not perform any analysis of any specific factors that could significantly alter or affect the treatment discontinuation rates. In fact, treatment discontinuation was associated with objective clinical data and provider factors, as well as demographic data and subjective symptoms in patients with LUTS. Thus, a large-scale, prospective, randomized trial should be performed to further investigate the factors influencing treatment discontinuation.

## 5. Conclusion

The discontinuation rate of tadalafil alone or in combination with ABs for LUTS with or without ED was relatively low and varied according to the study type. Patients treated with CT or monotherapy were similarly likely to abandon treatment. Furthermore, treatment with FDC appeared to have better persistence than the free-dose combination. These data may help guide clinicians in decision-making for drug regimens. At the same time, further studies are required to assess the factors affecting the discontinuation incidence and help develop strategies to reduce their occurrence.

## Figures and Tables

**Table 1:** Baseline characteristics of the included trials.

Study/Year	Country	Study design	Arm	Discontinuation (n)	Discontinuation due to AEs (n)	Discontinuation due to no efficacy (n)	Time of therapy (month)
McVary (2007) [18]	US	RCT	5 mg tadalafil (*N* = 138) 20 mg tadalafil (*N* = 129)	9 4	4 1	0 0	6

Roehrborn (2008) [5]	10 Countries^1^	RCT	2.5 mg tadalafil (*N* = 209) 5 mg tadalafil (*N* = 212) 10 mg tadalafil (*N* = 216) 20 mg tadalafil (*N* = 209)	27 30 41 47	4 12 11 14	1 2 1 2	12

Porst (2009) [19]	10 Countries^1^	RCT	2.5 mg tadalafil (*N* = 117) 5 mg tadalafil (*N* = 113) 10 mg tadalafil (*N* = 120) 20 mg tadalafil (*N* = 116)	16 16 23 27	3 6 6 6	0 1 0 2	12

Porst (2011) [20]	US, Argentina, Germany, Italy, and Mexico	RCT	5 mg tadalafil (*N* = 161)	13	3	1	12

Kim (2011) [21]	Korea	RCT	5 mg tadalafil (*N* = 51)	3	2	—	12

Oelke (2012) [22]	10 Countries^2^	RCT	5 mg tadalafil (*N* = 171)	15	2	0	12

Egerdie (2012) [23]	9 Countries^1^	RCT	2.5 mg tadalafil (*N* = 198) 5 mg tadalafil (*N* = 208)	26 24	3 6	1 3	12

Yokoyama (2012) [24]	Japan, Korea, and Taiwan	RCT	2.5 mg tadalafil (*N* = 151) 5 mg tadalafil (*N* = 155)	15 18	5 7	1 —	12

Takeda (2012) [25]	Japan	RCT OLE^3^	2.5 mg tadalafil (*N* = 142) 5 mg tadalafil (*N* = 140) 5 mg tadalafil (*N* = 394)	7 12 71	4 5 35	— 2 5	12 42

Takeda (2014) [6]	Japan and Korea	RCT	5 mg tadalafil (*N* = 306)	14	4	—	12

Yang (2019) [26]	Korean	RCT	5 mg tadalafil (*N* = 51)	7	2	—	12

Matsukawa (2019) [27]	Japan	Prospective	5 mg tadalafil (*N* = 105)	11	5	—	48

Bechara (2014) [28]	Argentina	Prospective	5 mg tadalafil (*N* = 67)	6	1	—	6

Zhang (2018) [29]	Mainland China, Taiwan and Korea	RCT	5 mg tadalafil (*N* = 363)	17	6	—	12

Bechara (2008) [30]	Argentina	RCT	20 mg tadalafil + 0.4 mg tamsulosin (*N* = 15)	1	1	—	6

Singh (2014) [31]	India	RCT	10 mg tadalafil (*N* = 44) 10 mg tadalafil + 0.4 mg tamsulosin (*N* = 44)	4 2	4 1	— —	12

Karami (2016) [32]	Iran	RCT	20 mg tadalafil (*N* = 61) 20 mg tadalafil + 0.4 mg tamsulosin (*N* = 61)	1 3	1 3	— —	12

Kim (2017) [33]	Korean	RCT OLE^3^	5 mg tadalafil (*N* = 171) 5 mg tadalafil + 0.2 mg tamsulosin (*N* = 170) 5 mg tadalafil + 0.4 mg tamsulosin (*N* = 169) 5 mg tadalafil + 0.4 mg tamsulosin (*N* = 443)	18 18 31 15	— — — —	— — — —	12 12

Nagasubramanian (2020) [34]	India	RCT	5 mg tadalafil + 0.4 mg tamsulosin (*N* = 69)	14	4	—	12

Lee (2012) [35]	Korea	Prospective	Tadalafil + ABs (tamsulosin or alfuzosin) (*N* = 158)	39	2	12	12

Ahn (2020) [36]	Korea	Retrospective	5 mg tadalafil + 0.4 mg tamsulosin (*N* = 97)	44	28	10	24

Wada (2020) [37]	Japan	Retrospective	Tadalafil (*N* = 155)	74	21	31	48

Pinggera (2014) [38]	US, Austria, and Italy	RCT	5 mg tadalafil (*N* = 47)	8	4	—	8

Takahashi (2020) [39]	Japan	Prospective	5 mg tadalafil (*N* = 31)	9	8	—	12

Matsukawa (2020) [40]	Japan	Prospective	5 mg tadalafil (*N* = 80)	11	4	—	48

Amano (2017) [41]	Japan	Prospective	5 mg tadalafil (*N* = 81)	4	1	1	24

Donatucci (2010) [42]	US and Canada	RCT	5 mg tadalafil (*N* = 427)	128	22	15	48

Choi (2014) [43]	Korea	Prospective	5 mg tadalafil (*N* = 90)	17	3	5	12

Kang (2012) [44]	Korea	RCT	5 mg tadalafil (*N* = 84)	18	3	1	12

Yoshida (2017) [45]	Japan	RCT	5 mg tadalafil (*N* = 139)	3	1	—	8

Urakami (2018) [46]	Japan	RCT	5 mg tadalafil (*N* = 38)	7	7	—	12

Takahashi (2016) [47]	Japan	Prospective	5 mg tadalafil (*N* = 35)	8	4	—	12

Liguori (2009) [48]	Italy	RCT	20 mg tadalafil (*N* = 21) 20 mg tadalafil + 10 mg alfuzosin (*N* = 23)	2 2	1 2	— —	12

Pattanaik (2019) [49]	India	RCT	5 mg tadalafil (*N* = 38)	1	—	1	6

Yamazaki (2020) [50]	Japan	Prospective	5 mg tadalafil (*N* = 1393)	672	98	130	72

Koyama (2018) [51]	Japan	Prospective	5 mg tadalafil (*N* = 58)	7	3	4	4

Madani (2012) [52]	Iran	RCT	10 mg tadalafil (*N* = 66)	6	6	—	12

Takeda (2017) [53]	Japan	RCT	5 mg tadalafil + 0.2 mg tamsulosin/4 mg silodosin (*N* = 167)	10	8	—	8

Goldfischer (2012) [54]	US	RCT	5 mg tadalafil + ABs^4^ (*N* = 158)	18	7	—	12

Morgia (2018) [55]	Italy	RCT	5 mg tadalafil (*N* = 136)	8	—	—	24

Dmochowski (2013) [56]	US and Canada	RCT	20 mg tadalafil (*N* = 99)	10	2	—	12

Dell'Atti (2015) [57]	Italy	RCT	5 mg tadalafil (*N* = 56)	3	3	—	12

Matsukawa (2021) [58]	Japan	Prospective	5 mg tadalafil (*N* = 66)	7	2	—	48

Maselli (2011) [59]	Italy	RCT	5 mg tadalafil (*N* = 28)	2	2	—	12

^1^Studies for which countries are unknown. ^2^The ten countries are Australia, Austria, Belgium, France, Germany, Greece, Italy, Mexico, the Netherlands, and Poland. ^3^Both these studies underwent two phases, namely, a double-blind period followed by an open-label extension phase. ^4^ABs here includes uroselective (alfuzosin, silodosin, or tamsulosin) and non-uroselective (doxazosin or terazosin).

**Table 2:** Results of subgroup analysis of the incidence of discontinuation with tadalafil alone or in combination with ABs for LUTS patients with or without ED.

Items	No. of studies	Discontinuation (n)	Index patients (n)	Incidence (%) (95% CI)	I^2^ (%)
*Drug regimen*					
Monotherapy	38	1527	7686	12.16 (9.20, 15.46)	97
CT	10	197	1547	12.87 (6.59, 20.86)	92
*Drug regimen (discontinuation due to AEs)*					
Monotherapy	36	361	7980	4.12 (3.08, 5.30)	79
CT	9	57	792	6.43 (2.72, 11.59)	84
*Study design*					
Prospective	11	791	2164	17.53 (10.87, 25.39)	97
Retrospective	2	118	252	46.82 (40.70, 52.99)	0
RCT	31	815	6844	9.78 (7.66, 12.12)	89
*Combination form*					
FDC	2	108	879	24.00 (0.52, 66.34)	99
FDC^*∗*^	1	64	782	8.18 (6.36, 10.33)	—
Free-dose combination	8	89	695	10.62 (5.84, 16.61)	81

^*∗*^Result after excluding the only retrospective study in this subset of FDC vs. free-dose combination. ABs: a-blockers; AEs: adverse events; CI: confidence interval; CT: combination therapy; ED: erectile dysfunction; FDC: fixed-dose combination; LUTS: lower urinary tract symptoms; RCT: randomized clinical trial.

## Data Availability

All data generated or analyzed during this study are included in this article (and its Supplementary material files).

## Supplementary Materials

Supplementary Materials Table S1: PRISMA 2020 checklist. Table S2: Search strategy. Table S3: Newcastle-Ottawa Scale assessment results of included observational studies. Table S4: Risk of bias of the RCTs calculated with the Cochrane risk of bias assessment tool. Figure S1: Forest plot illustrating subgroup analysis of the incidence of discontinuation with tadalafil alone or in combination with ABs for LUTS with or without ED. (A) Drug regimen, (B) Drug regimen (discontinuation due to AEs), (C) Study design, (D) Combination form. ABs: a-blockers; AEs: adverse events; CI: confidence interval; CT: combination therapy; ED: erectile dysfunction; FDC: fixed-dose combination; LUTS: lower urinary tract symptoms; RCT: randomized clinical trial. Figure S2: Cumulative meta-analysis of the discontinuation rate. CI: confidence interval. Figure S3: Sensitivity analysis of the discontinuation rate. CI: confidence interval.
