# Supplementary material for: Discontinuation Rates of Tadalafil Alone and in Combination with a-Blockers in the Treatment of Male Lower Urinary Tract Symptoms with or without Coexisting Erectile Dysfunction: A Systematic Review and Meta-Analysis
Source: Int J Clin Pract. 2022 Nov 3;2022:9298483. doi: 10.1155/2022/9298483 (PMC9649306; doi:10.1155/2022/9298483)
Supplement: Supplementary Materials — Table S1: PRISMA 2020 checklist. Table S2: Search strategy. Table S3: Newcastle-Ottawa Scale assessment results of included observational studies. Table S4: Risk of bias of the RCTs calculated with the Cochrane risk of bias assessment tool. Figure S1: Forest plot illustrating subgroup analysis of the incidence of discontinuation with tadalafil alone or in combination with ABs for LUTS with or without ED. (A) Drug regimen, (B) Drug regimen (discontinuation due to AEs), (C) Study design, (D) Combination form. ABs: a-blockers; AEs: adverse events; CI: confidence interval; CT: combination therapy; ED: erectile dysfunction; FDC: fixed-dose combination; LUTS: lower urinary tract symptoms; RCT: randomized clinical trial. Figure S2: Cumulative meta-analysis of the discontinuation rate. CI: confidence interval. Figure S3: Sensitivity analysis of the discontinuation rate. CI: confidence interval. [file 9298483.f1.docx]

**Table S1:** PRISMA 2020 checklist

| **Section and Topic** | **Item #** | **Checklist item** | **Location where item is reported** |
| --- | --- | --- | --- |
| **TITLE** | | |  |
| Title | 1 | Identify the report as a systematic review. | Page 1 |
| **ABSTRACT** | | |  |
| Abstract | 2 | See the PRISMA 2020 for Abstracts checklist. | Page 2 |
| **INTRODUCTION** | | |  |
| Rationale | 3 | Describe the rationale for the review in the context of existing knowledge. | Page 3 |
| Objectives | 4 | Provide an explicit statement of the objective(s) or question(s) the review addresses. | Page 3 |
| **METHODS** | | |  |
| Eligibility criteria | 5 | Specify the inclusion and exclusion criteria for the review and how studies were grouped for the syntheses. | Page 4 |
| Information sources | 6 | Specify all databases, registers, websites, organisations, reference lists and other sources searched or consulted to identify studies. Specify the date when each source was last searched or consulted. | Page 4 |
| Search strategy | 7 | Present the full search strategies for all databases, registers and websites, including any filters and limits used. | Page 4 |
| Selection process | 8 | Specify the methods used to decide whether a study met the inclusion criteria of the review, including how many reviewers screened each record and each report retrieved, whether they worked independently, and if applicable, details of automation tools used in the process. | Page 4 |
| Data collection process | 9 | Specify the methods used to collect data from reports, including how many reviewers collected data from each report, whether they worked independently, any processes for obtaining or confirming data from study investigators, and if applicable, details of automation tools used in the process. | Page 5 |
| Data items | 10a | List and define all outcomes for which data were sought. Specify whether all results that were compatible with each outcome domain in each study were sought (e.g. for all measures, time points, analyses), and if not, the methods used to decide which results to collect. | Page 4 |
|  | 10b | List and define all other variables for which data were sought (e.g. participant and intervention characteristics, funding sources). Describe any assumptions made about any missing or unclear information. | Page 4 |
| Study risk of bias assessment | 11 | Specify the methods used to assess risk of bias in the included studies, including details of the tool(s) used, how many reviewers assessed each study and whether they worked independently, and if applicable, details of automation tools used in the process. | Page 5 |
| Effect measures | 12 | Specify for each outcome the effect measure(s) (e.g. risk ratio, mean difference) used in the synthesis or presentation of results. | Page 5 |
| Synthesis methods | 13a | Describe the processes used to decide which studies were eligible for each synthesis (e.g. tabulating the study intervention characteristics and comparing against the planned groups for each synthesis (item #5)). | Page 5 |
|  | 13b | Describe any methods required to prepare the data for presentation or synthesis, such as handling of missing summary statistics, or data conversions. | Page 5 |
|  | 13c | Describe any methods used to tabulate or visually display results of individual studies and syntheses. | Page 5 |
|  | 13d | Describe any methods used to synthesize results and provide a rationale for the choice(s). If meta-analysis was performed, describe the model(s), method(s) to identify the presence and extent of statistical heterogeneity, and software package(s) used. | Page 5 |
|  | 13e | Describe any methods used to explore possible causes of heterogeneity among study results (e.g. subgroup analysis, meta-regression). | Page 6 |
|  | 13f | Describe any sensitivity analyses conducted to assess robustness of the synthesized results. | Page 5 |
| Reporting bias assessment | 14 | Describe any methods used to assess risk of bias due to missing results in a synthesis (arising from reporting biases). | Page 5 |
| Certainty assessment | 15 | Describe any methods used to assess certainty (or confidence) in the body of evidence for an outcome. | Page 5 |
| **RESULTS** | | |  |
| Study selection | 16a | Describe the results of the search and selection process, from the number of records identified in the search to the number of studies included in the review, ideally using a flow diagram. | Page 6 |
|  | 16b | Cite studies that might appear to meet the inclusion criteria, but which were excluded, and explain why they were excluded. | Page 6 |
| Study characteristics | 17 | Cite each included study and present its characteristics. | Page 6 |
| Risk of bias in studies | 18 | Present assessments of risk of bias for each included study. | Page 6-7 |
| Results of individual studies | 19 | For all outcomes, present, for each study: (a) summary statistics for each group (where appropriate) and (b) an effect estimate and its precision (e.g. confidence/credible interval), ideally using structured tables or plots. | Page 7-8 |
| Results of syntheses | 20a | For each synthesis, briefly summarise the characteristics and risk of bias among contributing studies. | Page 7-8 |
|  | 20b | Present results of all statistical syntheses conducted. If meta-analysis was done, present for each the summary estimate and its precision (e.g. confidence/credible interval) and measures of statistical heterogeneity. If comparing groups, describe the direction of the effect. | Page 7-8 |
|  | 20c | Present results of all investigations of possible causes of heterogeneity among study results. | Page 8 |
|  | 20d | Present results of all sensitivity analyses conducted to assess the robustness of the synthesized results. | Page 8 |
| Reporting biases | 21 | Present assessments of risk of bias due to missing results (arising from reporting biases) for each synthesis assessed. | Page 8 |
| Certainty of evidence | 22 | Present assessments of certainty (or confidence) in the body of evidence for each outcome assessed. | Page 8 |
| **DISCUSSION** | | |  |
| Discussion | 23a | Provide a general interpretation of the results in the context of other evidence. | Page 8-10 |
|  | 23b | Discuss any limitations of the evidence included in the review. | Page 10 |
|  | 23c | Discuss any limitations of the review processes used. | Page 10 |
|  | 23d | Discuss implications of the results for practice, policy, and future research. | Page 10 |
| **OTHER INFORMATION** | | |  |
| Registration and protocol | 24a | Provide registration information for the review, including register name and registration number, or state that the review was not registered. | Page 4 |
|  | 24b | Indicate where the review protocol can be accessed, or state that a protocol was not prepared. | Page 4 |
|  | 24c | Describe and explain any amendments to information provided at registration or in the protocol. | Page 4 |
| Support | 25 | Describe sources of financial or non-financial support for the review, and the role of the funders or sponsors in the review. | Page 1 |
| Competing interests | 26 | Declare any competing interests of review authors. | Page 11 |
| Availability of data, code and other materials | 27 | Report which of the following are publicly available and where they can be found: template data collection forms; data extracted from included studies; data used for all analyses; analytic code; any other materials used in the review. | Page 11 |

*From:*  Page MJ, McKenzie JE, Bossuyt PM, Boutron I, Hoffmann TC, Mulrow CD, et al. The PRISMA 2020 statement: an updated guideline for reporting systematic reviews. BMJ 2021;372:n71. doi: 10.1136/bmj.n71

**Table S2:** Search strategy

| PubMed | (("Lower Urinary Tract Symptoms"[MeSH Terms] OR "Lower Urinary Tract Symptoms"[Title/Abstract] OR ("Dysuria"[Title/Abstract] OR "Nocturia"[Title/Abstract] OR "Prostatism"[Title/Abstract] OR "urinary bladder overactive"[Title/Abstract] OR "urinary bladder underactive"[Title/Abstract]) OR "urinary incontinence"[Title/Abstract]) AND ("Tadalafil"[MeSH Terms] OR ("Adcirca"[Title/Abstract] OR "Cialis"[Title/Abstract] OR "Tadalafil"[Title/Abstract]))) AND (1000/1/1:2022/5/15[pdat]) (302) |
| --- | --- |
| Scopus | ( TITLE-ABS-KEY ( ( lower AND urinary AND tract AND symptom ) OR ( lower AND urinary AND tract AND symptom ) OR dysuria OR nocturia OR prostatism OR ( urinary AND bladder AND overactive ) OR ( urinary AND bladder AND underactive ) OR ( urinary AND incontinence ) ) ) AND ( TITLE-ABS-KEY ( adcirca OR cialis OR tadalafil ) ) (705) |
| Embase | (('lower urinary tract symptom'/exp) OR (lower:ab,ti AND urinary:ab,ti AND tract:ab,ti AND symptom:ab,ti OR dysuria:ab,ti OR nocturia:ab,ti OR prostatism:ab,ti OR (urinary:ab,ti AND bladder:ab,ti AND overactive:ab,ti) OR (urinary:ab,ti AND bladder:ab,ti AND underactive:ab,ti) OR (urinary:ab,ti AND incontinence:ab,ti))) AND (('tadalafil'/exp) OR (adcirca:ab,ti OR cialis:ab,ti OR tadalafil:ab,ti)) AND [01-01-1947]/sd NOT [16-05-2022]/sd AND [<1966-2022]/py (752) |
| Web of science | (TS=(lower urinary tract symptom) OR TS=(lower AND urinary AND tract AND symptom) OR TS=(dysuria OR nocturia OR prostatism) OR TS=(urinary AND bladder AND overactive) OR TS=(urinary AND bladder AND underactive) OR TS=( urinary AND Incontinence)) AND (TS=(adcirca OR cialis OR tadalafil)) Refined by: [excluding] Databases: ( MEDLINE ). Search language=English.   (260) |
| Cochrane Library | ((lower urinary tract symptom) OR (lower AND urinary AND tract AND symptom) OR (dysuria OR nocturia OR prostatism) OR (urinary AND bladder AND overactive) OR (urinary AND bladder AND underactive) OR ( urinary AND Incontinence)) AND (adcirca OR cialis OR tadalafil) Custom range: until 15/5/2022 (342) |

**Table S3:** Newcastle-Ottawa Scale assessment results of included observational studies

| **Author name:** **Matsukawa (2019)** | |
| --- | --- |
| **Items** | **Response options** |
| Selection | |
| *Representativeness of the exposed cohort* | *** |
| *Selection of the non exposed cohort* |  |
| *Ascertainment of exposure* | *** |
| *Demonstration that outcome of interest was not present at start of study* | *** |
| Comparability | |
| *Comparability of cohorts on the basis of the design or analysis* |  |
| Outcome | |
| *Assessment of outcome* | *** |
| *Was follow-up long enough for outcomes to occur* | *** |
| *Adequacy of follow up of cohorts* | *** |

| **Author name: Bechara (2014)** | |
| --- | --- |
| **Items** | **Response options** |
| Selection | |
| *Representativeness of the exposed cohort* | *** |
| *Selection of the non exposed cohort* |  |
| *Ascertainment of exposure* | *** |
| *Demonstration that outcome of interest was not present at start of study* | *** |
| Comparability | |
| *Comparability of cohorts on the basis of the design or analysis* |  |
| Outcome | |
| *Assessment of outcome* | *** |
| *Was follow-up long enough for outcomes to occur* | *** |
| *Adequacy of follow up of cohorts* | *** |

| **Author name:** **Lee (2012)** | |
| --- | --- |
| **Items** | **Response options** |
| Selection | |
| *Representativeness of the exposed cohort* | *** |
| *Selection of the non exposed cohort* | *** |
| *Ascertainment of exposure* | *** |
| *Demonstration that outcome of interest was not present at start of study* | *** |
| Comparability | |
| *Comparability of cohorts on the basis of the design or analysis* | ** |
| Outcome | |
| *Assessment of outcome* | *** |
| *Was follow-up long enough for outcomes to occur* | *** |
| *Adequacy of follow up of cohorts* | *** |

| **Author name:** **Ahn (2020)** | |
| --- | --- |
| **Items** | **Response options** |
| Selection | |
| *Representativeness of the exposed cohort* | *** |
| *Selection of the non exposed cohort* |  |
| *Ascertainment of exposure* | *** |
| *Demonstration that outcome of interest was not present at start of study* | *** |
| Comparability | |
| *Comparability of cohorts on the basis of the design or analysis* |  |
| Outcome | |
| *Assessment of outcome* | *** |
| *Was follow-up long enough for outcomes to occur* | *** |
| *Adequacy of follow up of cohorts* | *** |

| **Author name:** **Wada (2020)** | |
| --- | --- |
| **Items** | **Response options** |
| Selection | |
| *Representativeness of the exposed cohort* | *** |
| *Selection of the non exposed cohort* | *** |
| *Ascertainment of exposure* | *** |
| *Demonstration that outcome of interest was not present at start of study* | *** |
| Comparability | |
| *Comparability of cohorts on the basis of the design or analysis* | ** |
| Outcome | |
| *Assessment of outcome* | *** |
| *Was follow-up long enough for outcomes to occur* | *** |
| *Adequacy of follow up of cohorts* | *** |

| **Author name: Takahashi (2020)** | |
| --- | --- |
| **Items** | **Response options** |
| Selection | |
| *Representativeness of the exposed cohort* | *** |
| *Selection of the non exposed cohort* | *** |
| *Ascertainment of exposure* | *** |
| *Demonstration that outcome of interest was not present at start of study* | *** |
| Comparability | |
| *Comparability of cohorts on the basis of the design or analysis* | ** |
| Outcome | |
| *Assessment of outcome* | *** |
| *Was follow-up long enough for outcomes to occur* | *** |
| *Adequacy of follow up of cohorts* |  |

| **Author name: Matsukawa (2020)** | |
| --- | --- |
| **Items** | **Response options** |
| Selection | |
| *Representativeness of the exposed cohort* | *** |
| *Selection of the non exposed cohort* | *** |
| *Ascertainment of exposure* | *** |
| *Demonstration that outcome of interest was not present at start of study* | *** |
| Comparability | |
| *Comparability of cohorts on the basis of the design or analysis* | ** |
| Outcome | |
| *Assessment of outcome* | *** |
| *Was follow-up long enough for outcomes to occur* | * |
| *Adequacy of follow up of cohorts* | *** |

| **Author name: Amano (2017)** | |
| --- | --- |
| **Items** | **Response options** |
| Selection | |
| *Representativeness of the exposed cohort* | * |
| *Selection of the non exposed cohort* |  |
| *Ascertainment of exposure* | *** |
| *Demonstration that outcome of interest was not present at start of study* | *** |
| Comparability | |
| *Comparability of cohorts on the basis of the design or analysis* |  |
| Outcome | |
| *Assessment of outcome* | *** |
| *Was follow-up long enough for outcomes to occur* | *** |
| *Adequacy of follow up of cohorts* | *** |

| **Author name: Choi (2014)** | |
| --- | --- |
| **Items** | **Response options** |
| Selection | |
| *Representativeness of the exposed cohort* | *** |
| *Selection of the non exposed cohort* |  |
| *Ascertainment of exposure* | *** |
| *Demonstration that outcome of interest was not present at start of study* | *** |
| Comparability | |
| *Comparability of cohorts on the basis of the design or analysis* | **** |
| Outcome | |
| *Assessment of outcome* | *** |
| *Was follow-up long enough for outcomes to occur* | *** |
| *Adequacy of follow up of cohorts* | *** |

| **Author name: Takahashi (2016)** | |
| --- | --- |
| **Items** | **Response options** |
| Selection | |
| *Representativeness of the exposed cohort* | *** |
| *Selection of the non exposed cohort* |  |
| *Ascertainment of exposure* | *** |
| *Demonstration that outcome of interest was not present at start of study* | *** |
| Comparability | |
| *Comparability of cohorts on the basis of the design or analysis* |  |
| Outcome | |
| *Assessment of outcome* | *** |
| *Was follow-up long enough for outcomes to occur* | *** |
| *Adequacy of follow up of cohorts* |  |

| **Author name: Yamazaki (2020)** | |
| --- | --- |
| **Items** | **Response options** |
| Selection | |
| *Representativeness of the exposed cohort* | *** |
| *Selection of the non exposed cohort* |  |
| *Ascertainment of exposure* | *** |
| *Demonstration that outcome of interest was not present at start of study* | *** |
| Comparability | |
| *Comparability of cohorts on the basis of the design or analysis* |  |
| Outcome | |
| *Assessment of outcome* | *** |
| *Was follow-up long enough for outcomes to occur* | *** |
| *Adequacy of follow up of cohorts* | *** |

| **Author name: Koyama (2018)** | |
| --- | --- |
| **Items** | **Response options** |
| Selection | |
| *Representativeness of the exposed cohort* | *** |
| *Selection of the non exposed cohort* | *** |
| *Ascertainment of exposure* | *** |
| *Demonstration that outcome of interest was not present at start of study* | *** |
| Comparability | |
| *Comparability of cohorts on the basis of the design or analysis* | ** |
| Outcome | |
| *Assessment of outcome* | *** |
| *Was follow-up long enough for outcomes to occur* | *** |
| *Adequacy of follow up of cohorts* | *** |

| **Author name: Matsukawa (2021)** | |
| --- | --- |
| **Items** | **Response options** |
| Selection | |
| *Representativeness of the exposed cohort* | *** |
| *Selection of the non exposed cohort* | *** |
| *Ascertainment of exposure* | *** |
| *Demonstration that outcome of interest was not present at start of study* | *** |
| Comparability | |
| *Comparability of cohorts on the basis of the design or analysis* | ** |
| Outcome | |
| *Assessment of outcome* | *** |
| *Was follow-up long enough for outcomes to occur* | *** |
| *Adequacy of follow up of cohorts* | *** |

Observational studies with scores of 0-3, 4-6, 7-9 were, respectively, considered as low, moderate, and high quality.

**Table S4:** Risk of bias of the RCTs calculated with the Cochrane risk of bias assessment tool

| **Study/(year)** | **Random sequence**  **generation (selection bias)** | **Allocation concealment**  **(selection bias)** | **Blinding**  **of participants**  **and personnel**  **(performance**  **bias)** | **Blinding**  **of outcome**  **assessment**  **(detection**  **bias)** | **Incomplete**  **outcome**  **data**  **(attrition**  **bias)** | **Selective**  **reporting**  **(reporting**  **bias)** | **Other**  **bias** |
| --- | --- | --- | --- | --- | --- | --- | --- |
| McVary (2007) | Unclear | Unclear | Low (double-blind) | Low | Low | Low | Low |
| Roehrborn (2008) | Unclear | Unclear | Low (double-blind) | Low | Low | Low | Low |
| Porst (2009) | Unclear | Unclear | Low (double-blind) | Low | Low | Low | Low |
| Porst (2011) | Unclear | Unclear | Low (double-blind) | Low | Low | Low | Low |
| Kim (2011) | Unclear | Unclear | Low (double-blind) | Low | Low | Low | Low |
| Oelke (2012) | Unclear | Unclear | Low (double-blind) | Low | Low | Low | Low |
| Egerdie (2012) | Low (computer-generated random sequence) | Low (interactive voice response system) | Low (double-blind) | Low | Low | Low | Low |
| Yokoyama (2012) | Unclear | Unclear | Low (double-blind) | Low | Low | Low | Low |
| Takeda (2012)* | Unclear | Unclear | Low (double-blind) | Low | Low | Low | Low |
|  |  |  | High (open-label) |  |  |  |  |
| Takeda (2014) | Low (computer-generated random sequence) | Low (interactive voice response system) | Low (double-blind) | Low | Low | Low | Low |
| Yang (2019) | Low (computer-generated coding system) | Low (sealed and labeled medication packages) | Low (double-blind) | Low | Low | Low | Low |
| Zhang (2018) | Unclear | Unclear | Low (double-blind) | Low | Low | Low | Low |
| Bechara (2008) | Unclear | Unclear | Low (double-blind) | Low | Low | Low | Low |
| Singh (2014) | Low (computer-generated random table) | Unclear | High (open-label) | Low | Low | Low | Low |
| Karami (2016) | Unclear | Unclear | High (single-blind) | Low | Low | Low | Low |
| Kim (2017)* | Low (stratified block randomization method) | Low (Interactive Web Response System) | Low (double-blind) | Low | Low | Low | Low |
|  |  |  | High (open-label) |  |  |  |  |
| Nagasubramanian (2020) | Low (computer-generated block randomisation) | Low (sealed covers in containers) | Low (double-blind) | Low | Low | Low | Low |
| Pinggera (2014) | Unclear | Unclear | Low (double-blind) | Low | Low | Low | Low |
| Donatucci (2010) | Unclear | Unclear | High (open-label) | Low | Low | Low | Low |
| Kang (2012) | Low (computer random number generator) | Unclear | Unclear | Low | Low | Low | Low |
| Yoshida (2017) | Low (dynamic randomization algorithm) | Unclear | High (open-label) | Low | Low | Low | Low |
| Urakami (2018) | Low (a software program.) | Low (a central randomization system.) | Unclear | Low | Low | Low | Low |
| Liguori (2009) | Unclear | Unclear | High (open-label) | Low | Low | Low | Low |
| Pattanaik (2019) | Low (computer generated random numbers) | Low (sealed and labelled containers) | Low (double-blind) | Low | Low | Low | Low |
| Madani (2012) | Low (random method generated by Excel) | Unclear | Low (double-blind) | Low | Low | Low | Low |
| Takeda (2017) | Unclear | Low (interactive web response system) | Low (double-blind) | Low | Low | Low | Low |
| Goldfischer (2012) | Unclear | Unclear | Low (double-blind) | Low | Low | Low | Low |
| Morgia (2018) | Low (computer-generated sequence) | Unclear | High (open-label) | Low | Low | Low | Low |
| Dmochowski (2013) | Unclear | Unclear | Low (double-blind) | Low | Low | Low | Low |
| Dell’Atti (2015) | Unclear | Unclear | Unclear | Low | Low | Low | Low |
| Maselli (2011) | Unclear | Unclear | Low (double-blind) | Low | Low | Low | Low |

*Both this two studies went through two phases, namely: a double-blind period followed by an open-label extension phase.

(A) (B)

**
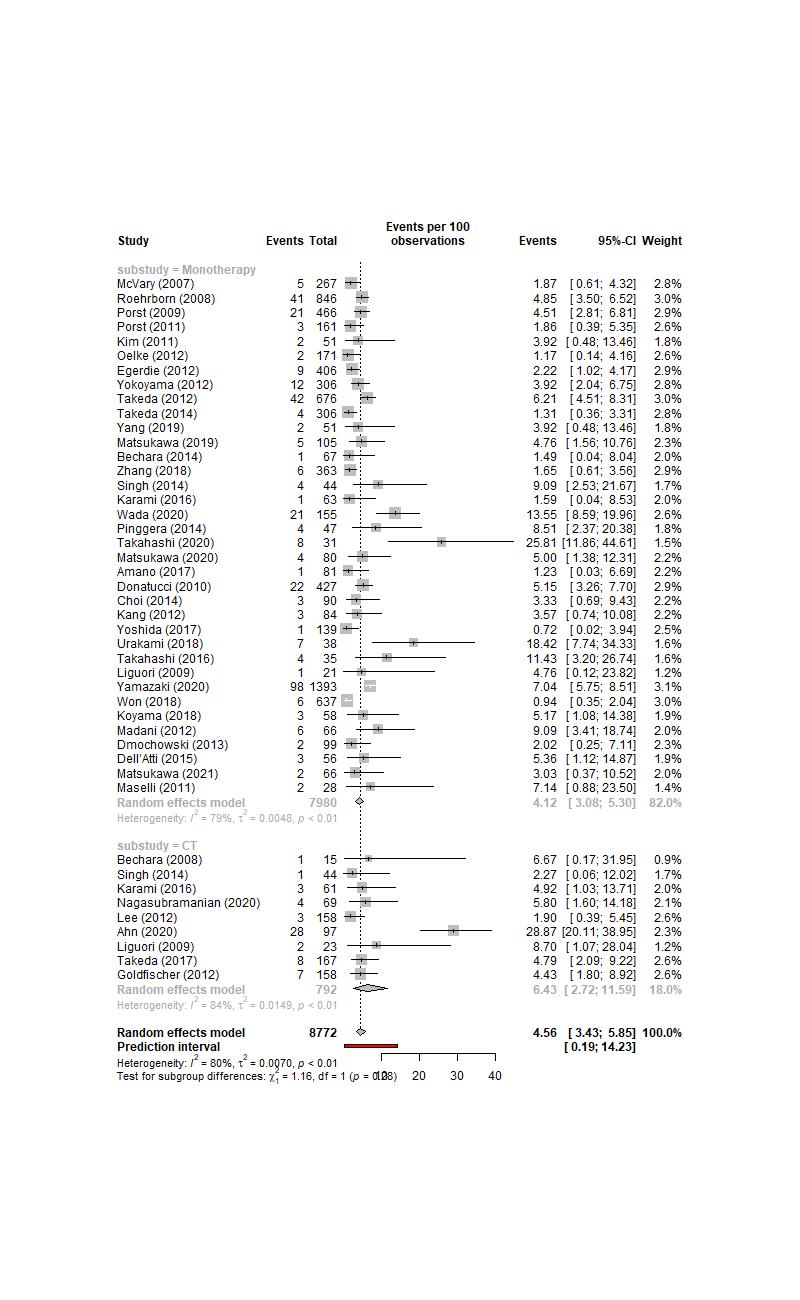

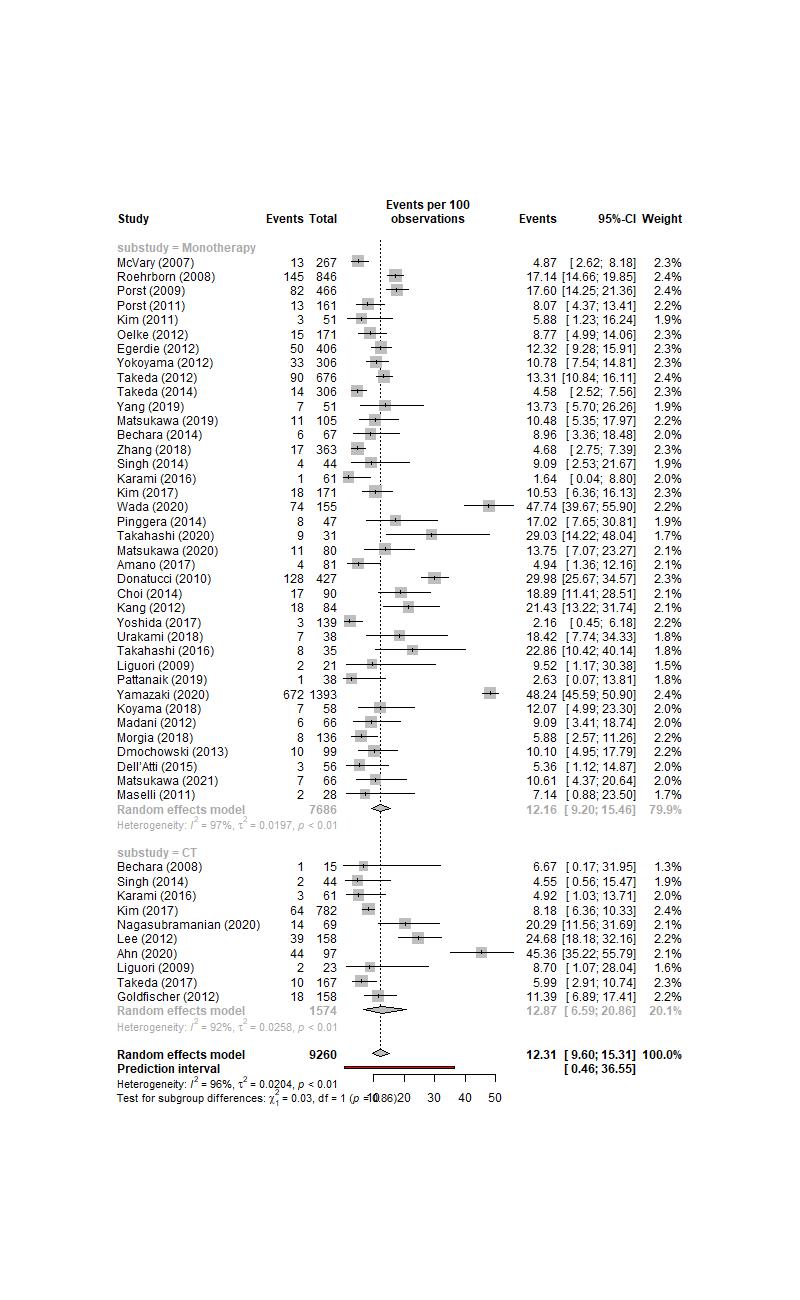
**

(C) (D)


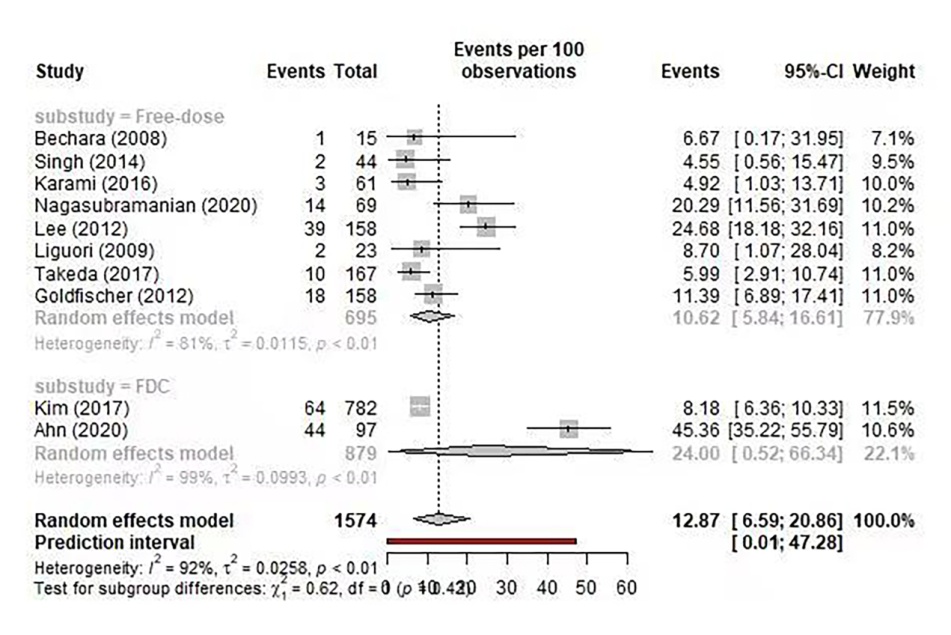

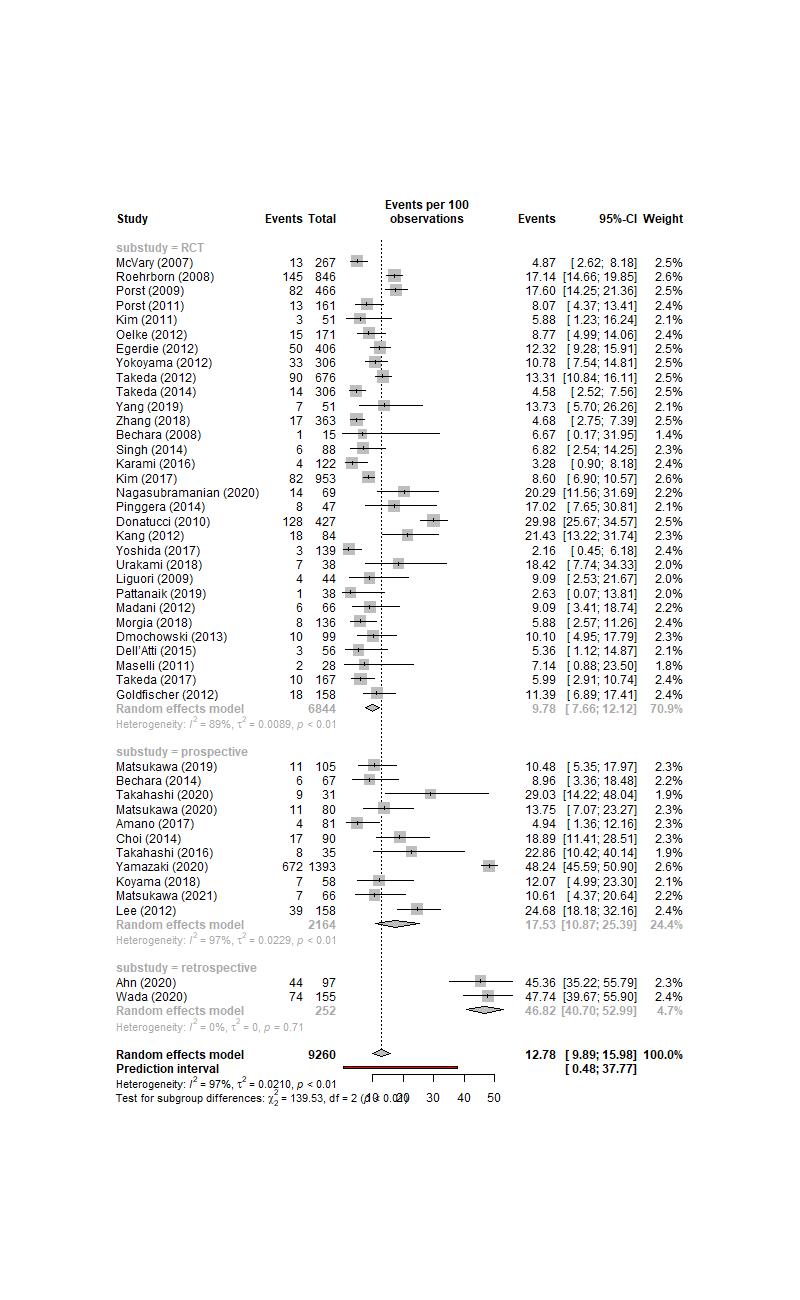


**Figure S1:** Forest plot illustrating subgroup analysis of the incidence of discontinuation with tadalafil alone or in combination with ABs for LUTS with or without ED. (A) Drug regimen, (B) Drug regimen (discontinuation due to AEs), (C) Study design, (D) Combination form. ABs: a-blockers; AEs: adverse events; CI: confidence interval; CT: combination therapy; ED: erectile dysfunction; FDC: fixed-dose combination; LUTS: lower urinary tract symptoms; RCT: randomized clinical trial.

**
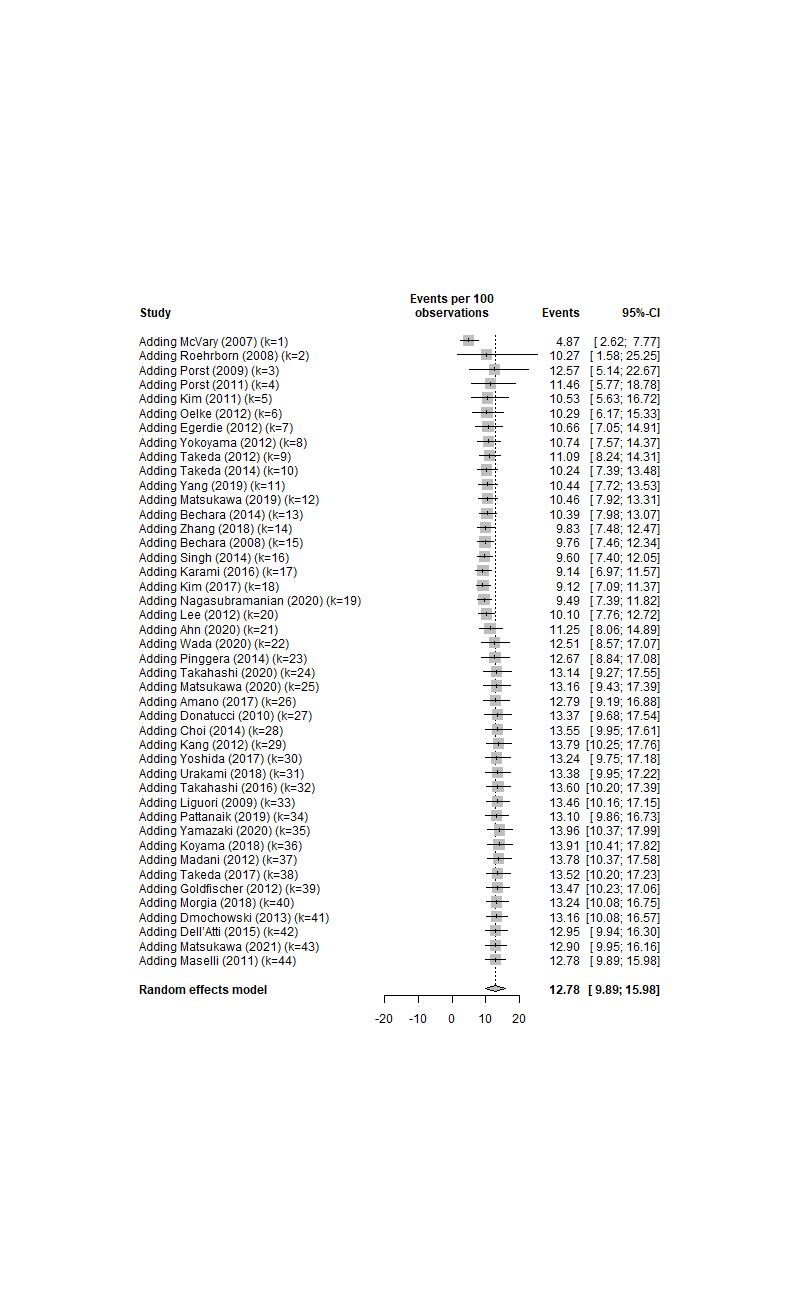
**

**Figure S2:** Cumulative meta-analysis of the discontinuation rate. CI: confidence interval.


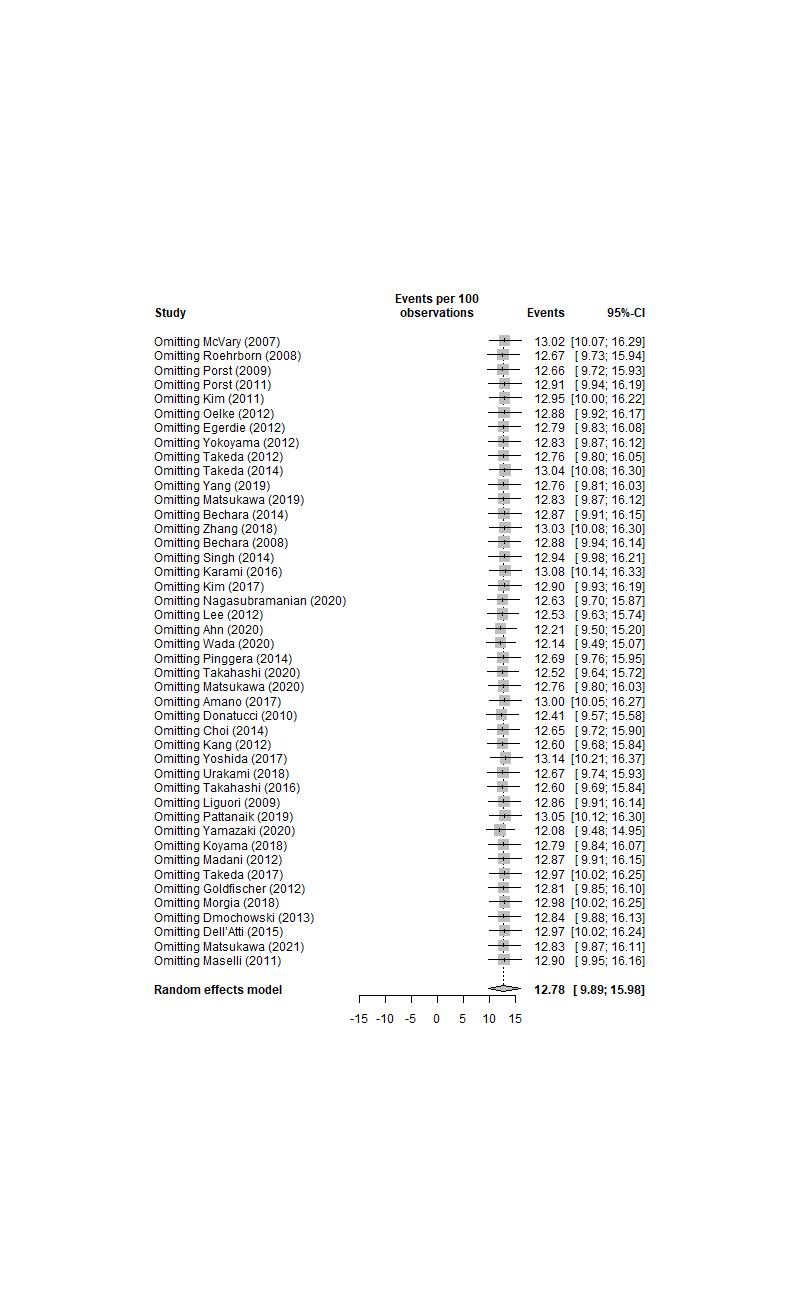


**Figure S3:** Sensitivity analysis of the discontinuation rate. CI: confidence interval.
